# Impact of Resolution of Hyponatremia on Neurocognitive and Motor Performance in Geriatric Patients

**DOI:** 10.1038/s41598-019-49054-8

**Published:** 2019-08-29

**Authors:** Paul Thomas Brinkkoetter, Franziska Grundmann, Panteha Jazayeri Ghassabeh, Ingrid Becker, Marc Johnsen, Victor Suaréz, Ralf-Joachim Schulz, Thomas Streichert, Volker Burst

**Affiliations:** 1 Department II of Internal Medicine and Center for Molecular Medicine Cologne, University of Cologne, Faculty of Medicine and University Hospital Cologne, Cologne, Germany; 2 Department of Geriatric Medicine, St. Marien-Hospital, Cologne, Germany; 3 Institute of Medical Statistics and Computational Biology, University of Cologne, Cologne, Germany; 4 Institute for Clinical Chemistry, University Hospital of Cologne, Cologne, Germany

**Keywords:** Endocrine system and metabolic diseases, Geriatrics, Neurological manifestations

## Abstract

This observational study investigated the impact of hyponatremia resolution on the results of a comprehensive geriatric assessment (CGA) in 150 patients with age ≥70 years and serum sodium <130 mEq/L. The test battery including Barthel index of Activities of Daily Living (ADL) and various tests of neurocognitive function, motor performance and mood stability was applied on admission and at discharge. Changes of individual test results (Δ) were analyzed and normonatremic patients matched for age, gender, and ADL served as reference group. Most CGA test results improved. The improvement was more pronounced in the hyponatremia group with respect to ADL (ΔADL: 14.3 ± 17.1 vs. 9.8 ± 14.7; p = 0.002) and MMSE (ΔMMSE: 1.8 ± 3.0 vs. 0.7 ± 1.9; p = 0.002). Effect sizes were small (i.e., >0.2) in the overall analysis for ΔADL and ΔMMSE and moderate (i.e., >0.5) for ΔMMSE in the euvolemic subgroup. Beneficial effects on ΔADL and ΔMMSE were only observed in the subgroup of patients in which [Na^+^] was raised by >5 mEq/L and multivariable linear regression analysis confirmed [Na^+^] increase to be an independent predictor of MMSE improvement. Resolution of hyponatremia has a beneficial impact on the geriatric patients’ overall functional status, in particular in euvolemic cases.

## Introduction

Hyponatremia is the most common disorder of water and electrolyte homeostasis. As prevalence increases with age, it adds considerably to the disease burden in geriatric patients^1–3^. The estimated mean prevalence for community-acquired hyponatremia ([Na^+^] <135 mEq/L) was 22.2% for patients on geriatric wards but only 6.0% for non-geriatric inpatients. Profound hyponatremia ([Na^+^] <125 mEq/L) was found more often with 4.5% vs. 0.8%^4^. Possible reasons for this include the kidney’s reduced capacity to produce a dilute urine with age, an accumulation of hyponatremia-associated comorbidities, and an increasing number of prescribed drugs that may induce hyponatremia^5^. Clinical symptoms are often subtle and interpreted as age-related. However, various analyses reported significant associations between hyponatremia and osteoporosis^6,7^, falls^8^, fractures^9,10^, delirious states^11^, cognitive impairment^12^, dementia^13^, and mortality^11^. To date it remains unclear whether hyponatremia plays a causative role and whether effective sodium control has a beneficial impact on these events. The value of to-date studies^14,15^ is limited due to small sample sizes and the use of assessments commonly not applied in the clinical setting.

The primary objective of this observational study was to analyze the impact of resolution of hyponatremia on the results of a commonly used comprehensive geriatric assessment (CGA) in a real-world setting.

## Results

**Figure 1:**
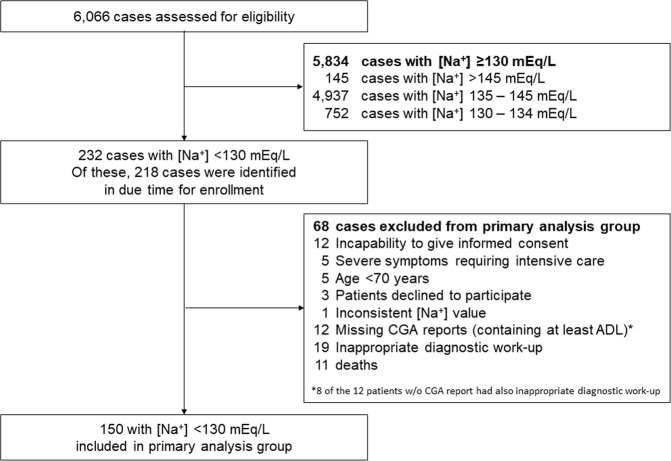
Study flow chart demonstrating patient enrollment.

In total, 6,066 geriatric patients were admitted during the study period. 145 (2.5%) were hypernatremic ([Na^+^] >145 mEq/L), 4,937 (81.5%) were normonatremic, and 984 (16%) were hyponatremic ([Na^+^] <135 mEq/L). 232 (3.8%) patients presented with a serum [Na^+^] <130 mEq/L, 14 of which were not identified by the study team in due time for enrollment, mainly due to the admission taking place on a weekend or holiday. 68 of the remaining 218 patients were either not eligible according to the pre-defined exclusion criteria or had to be excluded from the analysis because of missing ADL results, inconsistent sodium values (i.e., [Na^+^] ≥130 mEq/L on the day of the initial CGA) or inappropriate diagnostic work-up. In addition, 3 patients refused to participate, and 11 patients died while being hospitalized. The remaining 150 patients constitute the primary analysis group (Fig. 1).

Compared to a control group of 150 normonatremic patients matched only for age and gender the results of the test battery captured on admission indicated a significantly inferior performance in the primary analysis group in all domains except Geriatric Depression Scale (GDS). Similar results were observed in particular in the subgroup of patients with euvolemic hyponatremia. Analysis of test results of all 150 patients as well as of the subgroups with eu-, hypo-, and hypervolemic hyponatremia and the control group are shown in Supplemental Table 1.

In order to be able to perform a statistically sound analysis of the impact of hyponatremia treatment on the evolution of test results over time we sought to identify a second group, called reference group, that was matched for age, gender, and baseline test performance. Patients in the primary analysis group and in the reference group were well balanced with respect to most comorbidities and reasons for admission except osteoporosis and bone fractures. A standardized assessment of prevalent or historic geriatric syndromes revealed several between-group differences. Euvolemic, hypovolemic, and hypervolemic hyponatremia were present in 50%, 31% and 19%, respectively. The majority of patients received a monotherapy (64%) while 13.3% did not receive any hyponatremia-specific treatment. The median time interval between the two assessments was 17.5 (9–22) days in the hyponatremia and 17 (11–20) days in the reference group (p = 0.656). In the euvolemic subgroup, drugs (see Supplemental Material, Item S2) accounted for most cases of hyponatremia (44%). Nineteen patients (25%) were diagnosed with idiopathic SIADH. Three patients (4%) with a serum cortisol of <5 µg/dL (138nmol/L) suggestive of adrenal insufficiency were included here due to a lack of further diagnostic evaluation. In one patient with euvolemic hyponatremia, water intoxication due to tea-and-toast diet was confirmed. Patient demographics and clinical characteristics are depicted in Tables 1, 2.

**Table 1:** Demographics and patient characteristics.

	Hyponatremia (All) (n = 150)	Reference group (n = 150)	p
Age, [years]	82.5 (77–88)	82 (77–88)	0.648
Male [%]	25.3	25.3	
Comorbidities (%)			
Cardiovascular disease	141 (94)	136 (90.7)	0.405
Cancer	22 (14.7)	30 (20.0)	0.256
Arterial hypertension	138 (92)	132 (88)	0.307
Diabetes mellitus	36 (24)	49 (32.7)	0.117
Chronic kidney disease	56 (37.3)	63 (42)	0.450
Liver disease	9 (6)	2 (1.3)	0.065
Congestive heart failure	66 (44)	80 (53.3)	0.125
Neurologic/Psychiatric disorder	54 (36)	47 (31.3)	0.470
Pulmonary disease	23 (15.3)	30 (20)	0.337
Osteoporosis	48 (32)	79 (52.7)	<0.001
Reason for admission (%)			
Hyponatremia	5 (3.3)		
Bone fracture	27 (18)	43 (28.7)	0.029
Gait instability	49 (32.7)	41 (27.3)	0.374
Cardiovascular disease	11 (7.3)	10 (6.7)	1.000
Cancer	6 (4)	3 (2)	0.508
Infection	8 (5.3)	11 (7.3)	0.648
Impaired cognition	9 (6)	9 (6)	1.000
Congestive heart failure	12 (8)	9 (6)	0.664
Other	23 (15.3)	24 (16)	1.000
Geriatric syndromes (%)			
Immobility	136 (90.7)	148 (98.7)	0.004
Instability	145 (96.7)	147 (98)	0.727
Pain	112 (74.7)	110 (73.3)	0.897
Delirium	22 (14.7)	22 (14.7)	1.000
Dementia	61 (40.7)	57 (38)	0.712
Depression	58 (38.7)	29 (19.3)	0.001
Impaired hearing	70 (46.7)	47 (31.3)	0.005
Impaired vision	77 (51.3)	44 (29.3)	<0.001
Exsiccosis	27 (18)	13 (8.7)	0.024
Sarcopenia	53 (35.3)	25 (16.7)	<0.001
Dysphagia	21 (14)	11 (7.3)	0.076
Time between Tests [days]	17.5 (9–22)	17 (11–20)	0.656
[Na^+^] at 1. Test[mEq/L]	127 (124–129)	139 (137–141)	<0.001
[Na^+^] at 2. Test[mEq/L]	134 (131–137)	139 (137–142)	<0.001
Δ[Na^+^] [mEq/L]*	8 (5–13)	0 (0–0)	<0.001
[K^+^] at 1. Test [mEq/L]	4.2 (3.88–4.60)	4.3 (3.90–4.60)	0.786
[BG] at 1. Test [mg/dL]	99 (83.5–125)	101.5 (86–125)	0.979
[Hb] at 1. Test [g/dL]	11.1 (10.2–12.5)	10.8 (10.1–11–9)	0.226
	**Euvolemic Hyponatremia (n = 75)**	**Reference Group (n = 75)**	**p**
Age [years]	82 (77–88)	82 (77–87)	0.673
Male (%)	22.7	22.7	
Time between Tests (days)	17 (11–22)	17 (11–21)	0.833
[Na^+^] at 1. Test[mEq/L]	126 (123–128)	139 (138–141)	<0.001
[Na^+^] at 2. Test[mEq/L]	134 (131–137)	139 (138–142)	<0.001
Δ[Na^+^] [mEq/L]*	8 (5–13)	0 (0–0)	<0.001

*Δ[Na^+^]: Median change of serum [Na^+^] (IQR) from admission to discharge in the primary analysis group and euvolemic subgroup as well as their matched reference groups. [BG], blood glucose; [Hb], haemoglobin. Unless stated otherwise, numbers represent median (IQR).

**Table 2:** Hyponatremia severity, etiology and treatment in the primary analysis group and the euvolemic subgroup.

Severity of Hyponatremia	Hyponatremia, all (n = 150)	
	n (%)	Δ[Na^+^] [mEq/L]*
125 ≤[Na^+^] <130 (%)	103 (69)	7 (5–10)
120 ≤[Na^+^] <125 (%)	35 (23)	11 (6–14)
[Na^+^] <120 (%)	12 (8)	20 (13–21)
**Hyponatremia category**	**n (%)**	**Δ[Na** ^**+**^ ] [mEq/L]*
Euvolemic Hyponatremia	75 (50)	8 (5–13)
Hypovolemic Hyponatremia	47 (31)	9 (5–13)
Hypervolemic Hyponatremia	28 (19)	8 (5–11)
**Initial Therapy**	**n (%)**	**Δ[Na** ^**+**^ ] [mEq/L]*
No therapy	20 (13)	6.5 (2–10)
Monotherapy	96 (64)	9 (6–13)
Combination therapy	34 (23)	8 (5–11)
**Severity of Hyponatremia**	**Euvolemic Hyponatremia** (**n** = **75**)	
	**n (%)**	**Δ[Na** ^**+**^ ] [mEq/L]*
125 < = [Na^+^] < 130 (%)	48 (64)	6.5 (4–10)
120 < = [Na^+^] < 125 (%)	21 (28)	12 (6–14)
[Na^+^] < 120 (%)	6 (8)	20 (13–21)
**Etiology**	**n (%)**	**Δ[Na** ^**+**^ ] [mEq/L]*
Thiazide diuretic	17 (23)	8 (7–13)
Other Drugs	16 (21)	10.5 (6–15)
CNS disorder	15 (20)	6 (6–13)
Cancer	4 (5)	5 (4–10)
Pulmonary disorder	4 (5)	2.5 (2–5)
Idiopathic SIADH or other^§^	19 (25)	10 (5–15)
**Initial Therapy**	**n (%)**	**Δ[Na** ^**+**^ ] [mEq/L]*
No therapy	8 (11)	5.5 (0–11)
Monotherapy	54 (72)	8.5 (6–13)
Combination therapy	13 (17)	9 (6–14)
**Effectiveness of therapy**	**n (%)**	**Δ[Na** ^**+**^ ] [mEq/L]*
Drug withdrawal	36 (48)	10 (7–3)
Fluid restriction	21 (28)	9 (5–14)
Isotonic saline	14 (19)	7 (4–11)
Tolvaptan	5 (7)	7 (6–11)
Loop diuretic	4 (5)	5.5 (3–16)
Other	4 (5)	6 (3–19)

*Δ[Na^+^]: Median change of serum [Na^+^] (IQR) from admission to discharge in the primary analysis group and euvolemic subgroup.

^§^other includes: 1 case of tea-and-toast hyponatremia and 3 likely cases of adrenal insufficiency.

Unless stated otherwise, numbers represent median (IQR).

There were no between-group differences with respect to most components of the CGA upon admission, neither in the primary analysis group nor in the subgroups; a marginally significant difference (p = 0.045) was observed for the Timed Up and Go-Test (TuGT) in the hypovolemic group with a mean 33.00 s ± 18.44 s in the hyponatremia group vs. 19.64 s ± 7.81 s in the reference group (Supplemental Table 2). In both groups, a significant improvement over time was observed for Barthel index of ADL, Tinetti’s Performance Oriented Mobility Assessment (TPOMA), Timed Up and Go-Test (TuGT), evaluation of handgrip strength (HS), and Mini-Mental State Examination (MMSE) (Supplemental Table 3).

We calculated the numerical difference of the individual test results obtained on admission and at discharge in such a way, that a positive value would indicate an improvement. The improvements in ADL and MMSE were markedly more pronounced in the hyponatremic group as compared to their matched reference group with a mean ΔADL of 14.31 ± 17.12 vs. 9.84 ± 14.67 (p = 0.002) and a ΔMMSE 1.80 ± 3.00 vs. 0.67 ± 1.94 (p = 0.002), respectively (Table 3). In the euvolemic subgroup, a significantly larger increase in test results – reflecting superior improvement – was observed only for MMSE with a mean ΔMMSE of 2.08 ± 2.99 in hyponatremic patients vs. 0.55 ± 2.13 in the respective reference group (p = 0.001). A clear trend to a more pronounced improvement in ADL was found in all 3 subgroups as compared to their references, but significance was reached only in the hypervolemic patients (14.29 ± 15.50 vs. 8.04 ± 12.42, p = 0.049). There was a marginally significant change to the worse with regard to ΔETS (Esslinger Transfer Scale) in the euvolemic group and an improvement of the ΔTuGT results in the hypovolemic group (Table 3).

**Table 3:** Change (Δ) in test results between admission and discharge (positive values indicate an improvement) in hyponatremic patients of the primary analysis group as well as the euvolemic, hypovolemic, and hypervolemic subgroups vs. their respective matched reference groups, median (IQR) and mean ± SD.

	Hyponatremia	Reference group	n	p
**All patients**				
ΔADL	10 (0–25); 14.31 ± 17.12	5 (0–15); 9.84 ± 14.67	150	0.002
ΔTPOMA	4(1–8); 6.26 ± 9.54	4(0–7); 3.50 ± 5.82	50	0.061
ΔTuGT [s]	5 (1–7); 3.90 ± 14.68	5 (2–5); 4.58 ± 6.03	52	0.946
ΔHS [kg]	0 (0–3); 1.42 ± 6.55	0 (0–2); 0.91 ± 5.35	109	0.753
ΔMMSE	0 (0–3); 1.80 ± 3.00	0 (0–1); 0.67 ± 1.94	99	0.002
ΔGDS	0 (0–1); 0.09 ± 1.95	0 (0–0.25); −0.02 ± 2.11	68	0.957
ΔCDT	0 (0–0); −0.21 ± 1.19	0 (0–0); −0.21 ± 0.87	125	0.824
ΔETS	0 (0–1); 0.41 ± 0.97	0 (0–1); 0.54 ± 0.95	117	0.293
**Euvolemic subgroup**				
ΔADL	10 (0–25); 15.53 ± 18.50	5 (0–20); 11.27 ± 16.79	75	0.084
ΔTPOMA	4 (1.75–8); 5.78 ± 5.60	5 (1–9.50); 3.30 ± 6.49	23	0.205
ΔTuGT [s]	5 (0–5.5); 2.23 ± 9.48	5 (2–6); 5.18 ± 7.74	22	0.136
ΔHS [kg]	1 (0–4); 2.33 ± 6.46	0 (0–2); 1.36 ± 6.00	55	0.335
ΔMMSE	1 (0–3); 2.08 ± 2.99	0 (0–1); 0.55 ± 2.13	49	0.001
ΔGDS	0 (0–0); −0.26 ± 2.08	0 (0–0); −0.03 ± 2.52	31	0.353
ΔCDT	0 (0–0); −0.08 ± 1.26	0 (0–0); −0.15 ± 0.91	61	0.917
ΔETS	0 (0–1); 0.31 ± 0.91	0 (0–1); 0.67 ± 0.89	54	0.031
**Hypovolemic subgroup**				
ΔADL	10 (0–25); 12.38 ± 15.84	5 (0–10); 8.64 ± 12.11	47	0.071
ΔTPOMA	5 (3–8); 5.38 ± 2.94	2 (0–7); 3.00 ± 3.81	16	0.082
ΔTuGT [s]	5 (5–10); 10.39 ± 13.40	5 (2–5); 3.67 ± 1.94	18	0.020
ΔHS [kg]	0 (−0.5–2.5); 0.63 ± 5.63	0 (0–0.5); 0.09 ± 4.55	35	0.838
ΔMMSE	0 (0–2); 1.72 ± 3.45	0 (0–3); 0.83 ± 2.09	29	0.476
ΔGDS	0 (0–1); 0.1304 ± 1.89	0 (0–1); −0.09 ± 2.17	23	0.825
ΔCDT	0 (0–0); −0.47 ± 1.31	0 (−1–0); −0.32 ± 1.02	38	0.501
ΔETS	1 (0–1); 0.51 ± 1.12	0 (0–1); 0.36 ± 1.18	39	0.517
**Hypervolemic subgroup**				
ΔADL	10 (0–20); 14.29 ± 15.50	2.5 (0–13.75); 8.04 ± 12.42	28	0.049
ΔTPOMA	2.5 (0–4); 8.55 ± 18.88	3 (0–6); 4.64 ± 7.06	11	0.671
ΔTuGT [s]	5 (−3–6.25); −2.75 ± 20.65	4.5 (0–5); 4.83 ± 6.82	12	0.507
ΔHS [kg]	0 (−1–0); 0.26 ± 8.21	0 (0–2); 1.11 ± 4.76	19	0.348
ΔMMSE	0 (0–3); 1.24 ± 2.30	0 (0–1.25); 0.71 ± 1.15	21	0.303
ΔGDS	0 (0–1); 0.79 ± 1.62	0 (0–0); 0.14 ± 0.36	14	0.131
ΔCDT	0 (0–0); −0.12 ± 0.77	0 (0–0); −0.19 ± 0.49	26	0.565
ΔETS	0 (0–1): 0.46 ± 0.83	0 (1–1); 0.54 ± 0.59	24	0.800

Numbers are dimensionless except ΔTuGT (s) and ΔHS (kg).

**Figure 2:**
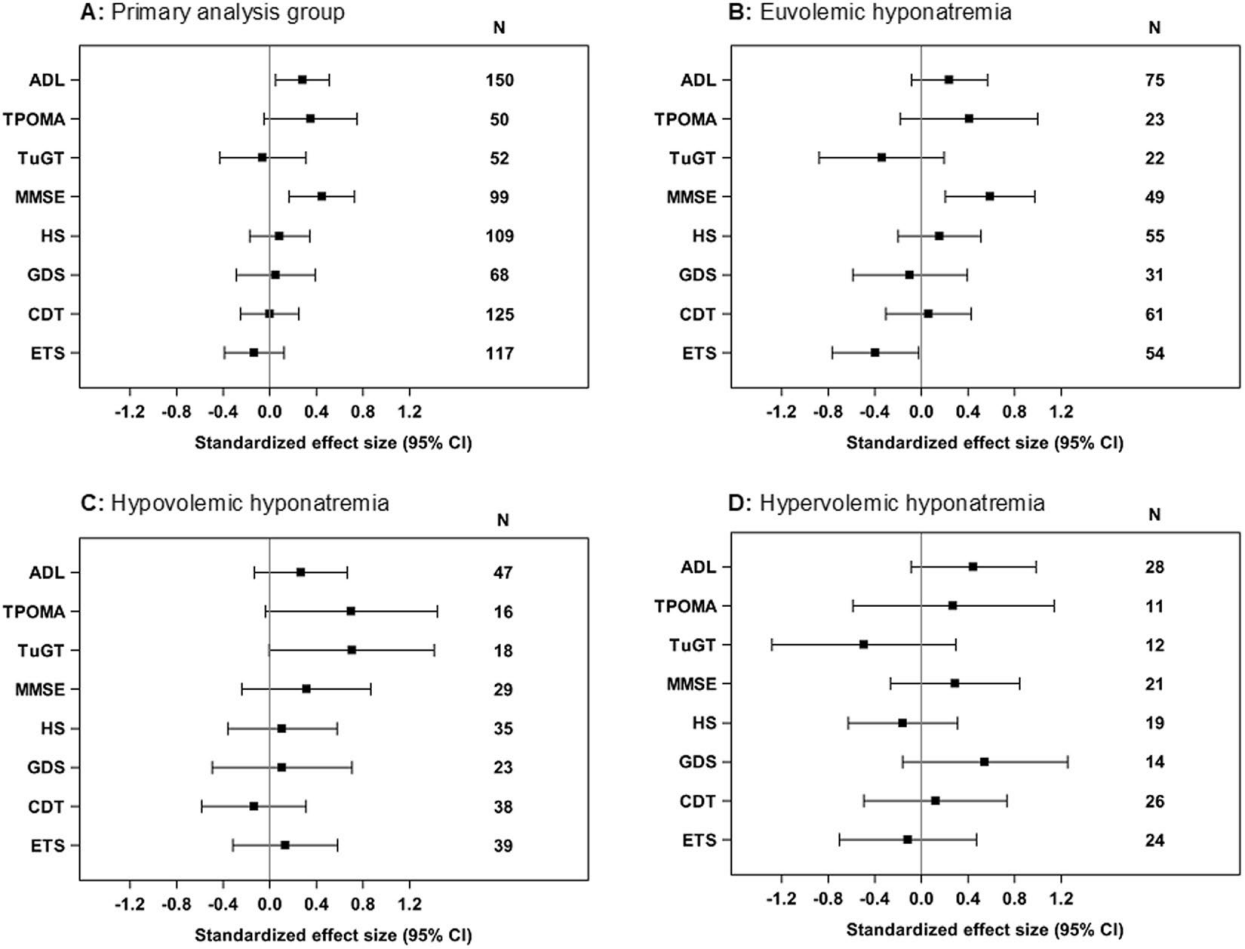
Standardized treatment effect sizes (95% CI) on the main outcome: changes of test results from admission to discharge in the primary analysis group (**A**) and the subgroups with euvolemic (**B**), hypovolemic (**C**), and hypervolemic (**D**) hyponatremia in comparison to their respective matched reference groups. Positive values indicate a greater improvement in the hyponatremia groups over reference group.

With 0.28 for ΔADL and 0.45 for ΔMMSE, effect sizes indicating a beneficial impact of hyponatremia resolution were small in the primary analysis group while a moderate effect size was observed in the euvolemic subgroup with 0.59 for ΔMMSE and in the hypovolemic subgroup with 0.70 for ΔTuGT^16^. With −0.39 the effect size for the worsening of the ETS results in the euvolemic subgroup was small, too (Fig. 2).

**Figure 3:**
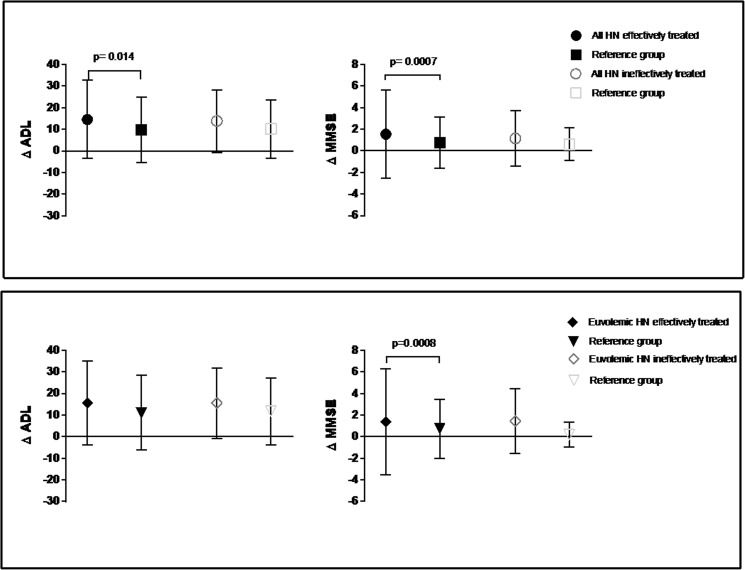
Mean change (±SD) in ADL (ΔADL) and MMSE (ΔMMSE) between admission and discharge in effectively treated (i.e., Δ[Na^+^] >5mEq/L, n = 108) and ineffectively treated (i.e., Δ[Na^+^] ≤5mEq/L, n = 42) hyponatremic patients of the primary analysis group (upper panel) and the euvolemic subgroup (lower panel) vs. their respective matched reference groups. Positive values indicate an improvement in test performance.

In the primary analysis group, a significant improvement in ΔADL and ΔMMSE was only seen in the effectively treated patients (i.e., Δ[Na^+^] >5 mEq/L, n = 108) with 14.51 ± 18.08 vs. 9.73 ± 15.91 (p = 0.014) and 1.67 ± 4.31 vs. 0.81 ± 2.17 (p = 0.007), respectively, but not in the ineffectively treated patients (i.e., Δ[Na^+^] ≤5 mEq/L). The same was observed for ΔMMSE with 1.53 ± 5.22 vs. 0.83 ± 2.52 (p = 0.008) in the euvolemic hyponatremia group, even after removing cases of drug-induced hyponatremia from the analysis (Fig. 3, Supplemental Table 4a–e). When the primary analysis group was split into a subgroup with moderate hyponatremia (i.e., 125 mEq/L ≤ [Na^+^] <130 mEq/L, n = 103) and a subgroup with profound hyponatremia (i.e., [Na^+^] <125 mEq/L) at baseline, significant differences were seen only in the moderate hyponatremia group with respect to ΔADL (p = 0.011) and ΔMMSE (p < 0.001) although the median [Na^+^] increase was significantly larger in the group with profound hyponatremia (13 (7–15) mEq/L vs. 7 (5–10) mEq/L; p < 0.0001) (Supplemental Table 5).

Linear regression analysis confirmed Δ[Na^+^] to be an independent predictor of ΔMMSE in the primary analysis group (Table 4), however, with p = 0.084 the significance level was not reached in the euvolemic subgroup. With respect to ADL, curve-fitting suggested a cubic rather than a linear relationship between Δ[Na^+^] and ΔADL revealing a possible biphasic pattern showing a positive correlation with a Δ[Na^+^] ≤10 mEq/L and a negative correlation with a Δ[Na^+^] >10 mEq/L. Consistent with the between-group analysis above, no meaningful relationships were detected between Δ[Na^+^] and any of the other CGA tests.

**Table 4:** Multivariable linear regression analysis of the determinants of the change of MMSE results between admission and discharge in the primary analysis group (n = 110) and the euvolemic subgroup (n = 56).

Primary analysis group, *R*^2^ = 0.274	B	Std. Error	CI	p
Age, year	0.021	0.040	−0.058–0.099	0.606
Gender, male	−0.077	0.695	−1.455–1.302	0.912
[Na^+^] at baseline, mEq/L	0−141	0.080	−0.017–0.299	0.080
Δ[Na^+^], mEq/L	0.179	0.067	0.047–0.312	0.009
MMSE at baseline	−0.222	0.053	−0.327–0.116	<0.001
Anemia at baseline	0.710	0.592	−0.465–1.886	0.233
Δ[Hb], g/dL	−0.119	0.190	−0.496–0.258	0.534
Occurrence of hypoglycemic episode	−0.401	0.732	−1.854–1.053	0.586
History of delirium	−1.419	0.769	−2.945–0.107	0.068
Number of comorbidities	0.113	0.198	−0.279–0.506	0.568
**Euvolemic group**, ***R***^***2***^ = **0**.**167**	**B**	**Std**. **Error**	**CI**	**p**
[Na^+^] at baseline, mEq/L	0.126	0.110	−0.095–0.346	0.258
Δ[Na^+^], mEq/L	0.171	0.097	−0.024–0.366	0.084
MMSE at baseline	−0.217	0.078	−0.373–−0.062	0.007
History of delirium	−1.371	0.981	−3.341–0.600	0.169

Δ[Na^+^], change of serum sodium between admission and discharge; Δ[Hb], change of hemoglobin between admission and discharge.

Due to small group size the number of potential predictors were reduced in the model for the euvolemic group and included only those variables that were found to be significant in the model for the primary analysis group.

Finally, we evaluated the reference limits for [Na^+^] in our local geriatric population using an indirect approach. The lower reference limit for [Na^+^] was estimated to be 134.9 mEq/L, 134.3 mEq/L, 134.5 mEq/L, 133.9 mEq/L, and 133.5 mEq/L for the age groups 50–59 years, 60–69 years, 70–79 years, 80–89 years, and 90–99 years, and gender-specific differences were clinically not relevant (Supplemental Material, Item S3). Using these new reference limits we re-counted the number of hyponatremic patients to be only 743 (12%).

## Discussion

This study demonstrates the effect of inpatient care in a specialized geriatric institution on the results of a CGA test battery in patients admitted with or for non-severely symptomatic hypotonic hyponatremia. As a major finding, the improvement in ADL and MMSE was significantly more pronounced in the hyponatremia group as compared to a normonatremic reference group matched for age, gender, and baseline test results. While there was a clear trend towards improvement in ADL in all hyponatremia subgroups, the improvement seen with MMSE results was largely driven by the euvolemic subgroup. Moreover, the calculated effect sizes suggest that these improvements – albeit numerically small – have to be considered clinically relevant as defined by Cohen’s criteria^16^. Assuming that medical efforts and specific geriatric rehabilitation measures were carried out identically in both groups, these differences are most likely accounted for, at least in part, by the resolution of hyponatremia itself. Moreover, while findings in the hypovolemic and the hypervolemic subgroups may be attributed to recovery from volume depletion or successful decongestion, respectively, rather than to hyponatremia resolution itself, the results obtained from the analysis of the euvolemic subgroup infer that effectively reversing hyponatremia indeed has a measurable beneficial impact on neurocognitive function and patients’ capability to recover or retain independence from permanent care. This is further supported by two findings. Firstly, the beneficial effect on MMSE in the euvolemic subgroup persisted after we had removed those patients from the analysis in whom drugs had been discontinued that are known to be possible inducers of hyponatremia (either thiazides and/or other hyponatremia-inducing drugs) but that might also have an intrinsic impact on CGA results by themselves. Secondly, multivariable linear regression analysis clearly confirmed sodium increase as an independent predictor of MMSE improvement. Consistently, in the between-group analysis the improvement in ADL and MMSE was only observed in those patients in whom the [Na^+^] had been increased by more than 5mEq/L, a threshold that is commonly accepted as treatment success.

Two studies have analyzed the effect of hyponatremia treatment on clinical outcomes. The *INSIGHT* study investigated the impact of effective [Na^+^] control with tolvaptan on the results of a test battery comprising pre-specified neurocognitive domains in elderly subjects^15^. Although a trend towards improvement with hyponatremia treatment was detected, only the results of the Morse tapping test assessing the psychomotor speed domain were statistically significant. Renneboog *et al*. evaluated neurocognitive function by analyzing the response time to visual and auditory stimuli and found a highly significant reduction of the average latency by 8.6% when the patients were rendered normonatremic^14^. With an estimated Cohen’s d of 0.32, the effect sizes are similar to the findings reported in the *INSIGHT* trial. Despite the differences in the test batteries applied in these trials and our study, they are consistent in demonstrating a beneficial effect of hyponatremia treatment on neurocognitive function. Although these effects are small with short-term treatment over days and weeks, the long-term impact might well be larger. Given two longitudinal analyses showing that hyponatremia is associated with prevalent cognitive impairment but also with future cognitive decline^17^ and development of dementia^13^ this would be of eminent clinical importance.

No significant effects were detectable with respect to motor performance in our study. In contrast, Renneboog and colleagues showed a reversible impairment of gait stability using a pressure-sensitive platform in mild-to-moderate hyponatremic patients often claimed to be responsible for a higher incidence of falls and fractures in this population^18–21^. To our knowledge, these analyses were not adjusted for the patients’ general medical condition and respective reference groups were – if at all – matched only for age and gender. However, since baseline performance is likely to impact on test evolution – e.g. baseline MMSE was shown to be a significant independent factor in linear regression in this analysis – we sought to improve statistical quality by adjusting for initial test results. This was achieved by adding ADL – a validated multi-domain-integrating measure of overall fitness – as match criterion. Of note, significantly more patients were admitted with bone fractures and pre-existing osteoporosis in the so-obtained reference group than in the hyponatremic patient group. Although speculative, this finding suggests that it might be the extent of frailty rather than hyponatremia itself that is associated with these adverse motor events.

Although the primary goal of this study was to analyze the effect of hyponatremia treatment on neurocognitive and motor performance over time, we also looked at the effect of hyponatremia on CGA itself by comparing the baseline test results of hyponatremic patients with an age- and gender-matched control group that was not adjusted for ADL or any other baseline test result. In line with published reports^12,17^, we observed a significant and clinically meaningful association of hyponatremia with inferior test results strongly supporting the notion of a true impact of hyponatremia on neurocognitive and possibly motor function.

The fact that the beneficial effects seen with ADL and MMSE were not reproducible in other tests in our analysis suggests that so far we have not found the appropriate test yet. In turn, this also signifies that our understanding of the precise impact of hyponatremia on cerebral functions is far from being complete.

Given the reported high incidence of hyponatremia in older subjects, we scrutinized whether the commonly used reference limits for serum sodium which are derived from healthy young adults also apply to our geriatric population. Overseeing data from more than 110,000 patients, McKee and co-workers calculated that the mean [Na^+^] was lower and the range wider in hospitalized patients >65 years^22^. We applied an indirect method which allows the determination of reference levels from data of a mixed population of healthy and diseased individuals^23^. Despite the postulated age-related reduction of the kidney’s capacity to produce dilute urine, the estimated lower reference level even for very old people was only slightly lower than the accepted value of 135 mEq/L and there were no gender-related differences. These findings imply that even mild-to-moderate degree hyponatremia must not be mistaken as a normal-for-age variation. However, with a prevalence of 12% applying the newly estimated reference limits instead of 16%, the reported age-related increase of hyponatremia rate might be questionable or at least less dramatic.

Several limitations apply, some of which arise from the observational case-control design in a real-life setting. Although this is the study with the largest sample size looking at treatment impact so far, the numbers of some individual test results were considerably smaller than 150, which had an impact on the power of statistical analysis. Notwithstanding, numbers for ADL and MMSE were high enough to allow for robust statistic analyses. Moreover, tests that a patient was unable to perform on admission but in which he did well on discharge were not included in the analysis. Hence, our findings most probably underestimate a true favorable effect of hyponatremia resolution.

As a matter of fact, case-control studies certainly cannot prove a true causative relationship with certainty. It must also be acknowledged that improvement in a test result might be secondary to treatment of the primary disease that caused the hyponatremia rather than to treatment of hyponatremia itself. However, since randomized controlled trials investigating the effect of hyponatremia treatment on outcome are cumbersome or even impossible to conduct for various practical as well as ethical reasons, observational studies with matched control groups provide the best possible source of evidence. In the geriatric patient cohort, any matching algorithm is always severely hampered by the multimorbidity of these patients making it almost impossible to find a perfectly matched reference group to serve as control. Here, we undertook the effort to identify well-matched patients to serve as reference group by screening a timespan of almost 4 years. Notwithstanding, there were some between-group discrepancies, in particular with respect to prevalent or historic geriatric syndromes. Without doubt, these differences may also have had an impact on the CGA results and, ultimately, the 1:1 matching strategy may not have provided representative data.

Further drawbacks of our study include the inability to distinguish cases of acute and chronic hyponatremia, and missing detailed information on chronic medication and the circumstances of living of the patients (e.g., were they admitted from nursing homes, etc.), features that may have had an impact on neurocognitive function.

In conclusion, there is a growing body of evidence that the resolution of hyponatremia in a geriatric population has a beneficial impact on neurocognitive function and possibly motor performance suggesting a true causative relationship. We demonstrate that successful sodium control improves Barthel index of ADL, an easy-to-apply multi-domain test capturing functional disabilities, which potentially translates into enhanced quality of life^24^ as well as MMSE performance. Future studies analyzing the mid- and long-term effects of hyponatremia therapy are warranted to fully appreciate the impact on frail old people.

## Methods

### Study design and participants

This study was designed at the University Hospital Cologne, approved by the Ethics Committee of the University of Cologne (reference number: 14–064) and conducted as a single-center, observational study at the Department of Geriatric Medicine at a community hospital in Cologne (St. Marien-Hospital), Germany. Between April 1^st^ 2014 and March 31^st^ 2016, all patients with a blood glucose-corrected serum [Na^+^] <130 mEq/L (Beckman Coulter AU 5800 Clinical Chemistry Analyzer, Inc. Brea, CA, USA) on admission were identified. Exclusion criteria comprised the presence of severe symptoms on admission necessitating intensive care support, age <70 years, and the incapability to give informed consent. We aimed for a primary analysis dataset of 150 patients with documented CGA containing valid data of the Barthel index of Activities of Daily Living and at least one of the other tests included in the CGA on admission and at discharge. Informed consent was obtained from all participants. Two retrospectively identified sets of patients who were admitted to the same institution between April 1^st^, 2014 and December 31^st^, 2017 with a [Na^+^] between 135 mEq/L and 145 mEq/L and who were matched (1:1) either for age (within 1 year), gender, and ADL on admission or for age and gender only served as so-called reference and control group, respectively.

Therapeutic measures – in particular treatment of hyponatremia – and specialized geriatric care were carried out entirely by the treating physicians and staff, independently from the investigators. Technicians performing CGA were not involved in treatment of hyponatremia to minimize bias. The study was conducted in accordance with the Declaration of Helsinki and the GCP guidelines endorsed by the International Conference on Harmonization. The study was registered on www.clinicaltrials.gov (NCT02242604).

### Procedures

After obtaining informed consent additional laboratory tests including serum creatinine, urea, uric acid, osmolality, cortisol, thyroid-stimulating hormone and urinary sodium, potassium, creatinine, urea, uric acid and osmolality were performed in order to accurately assess the etiology of hyponatremia. Protocol-specified criteria for assessing volume status included evidence of peripheral edema, evidence of pulmonary edema, evidence of ascites, evidence of raised jugular venous pulse, and evidence of exsiccosis (dry mucous membranes, reduced skin turgor). All patients were evaluated by 2 out of 3 experienced nephrologists (V.B., F.G., P.T.B.).

### Comprehensive geriatric assessment

The CGA is a multi-dimensional diagnostic and therapeutic process to determine the medical, mental, and functional disabilities of frail older people^25^. It utilizes a standardized set of tests; i.e. Barthel index of ADL^26^, Tinetti’s Performance Oriented Mobility Assessment (TPOMA)^27^, Timed Up and Go-Test (TuGT)^28^, Mini-Mental State Examination (MMSE)^29^, evaluation of handgrip strength (HS)^30^, Geriatric Depression Scale (GDS)^31^, Clock-drawing Test (CDT)^32^, and Esslinger Transfer Scale (ETS)^33^. A more detailed description is provided in the Supplemental Material (Item S1). As a mandatory part of the clinical routine in this geriatric department the CGA is performed on the day of or – if admitted on a weekend – within 48 hours after admission and on the day of discharge in every patient admitted to this institution by specialized technicians. Technicians were not involved in treatment of hyponatremia. Sodium values assessed on the same day on which the CGA was performed were used for analysis and patients were excluded from the analysis if the [Na^+^] value on the day of the first CGA was ≥130 mEq/L.

### Outcomes

The main outcome parameter was difference between the two groups in the change of individual CGA results from admission to discharge. We calculated the numerical difference (Δ) for each individual test in such a way, that a positive value would indicate an improvement.

### Statistical analysis

Given the exploratory nature of this study, no sample size calculation and no adjustments for multiple testing were performed. Descriptive statistics are provided for categorical variables as frequencies and compared using McNemar’s test. Group comparisons of numerical data were conducted using Wilcoxon signed-rank test. All results are given as median (interquartile range, [IQR]) or mean ± standard deviation (SD). Cohen’s d with its 95% confidence interval (CI) is provided as an estimate of the effect size. Concerning missing data, pairwise deletion, and no imputation was performed. Possible associations between the change of each CGA result (ΔCGA) and the change of [Na^+^] (Δ[Na^+^]) were evaluated applying multivariable linear regression analysis with age, gender, baseline [Na^+^], baseline CGA, anemia (i.e., hemoglobin <12 g/dL in women and <13 g/dL in men) on admission, change of hemoglobin (Δ[Hb]) during stay, occurrence of hypoglycemic episodes during stay (i.e., blood glucose <70 mg/dL), past episodes of delirium, and number of documented comorbidities as independent variables. In order to avoid artificial non-linearity, those cases that scored top results in each CGA test (e.g., 100 in ADL, or 30 in MMSE) at discharge were excluded from the regression analysis. Two-sided p values <0.05 were considered statistically significant. Age-related reference range limits of sodium for the general population of Cologne were assessed by applying an indirect method^23,34^. Analysis software was IBM SPSS Statistics Version 24 and SAS 9.3, SAS Institute Inc., Cary, USA.

## Supplementary information

Supplementary Material

## Data Availability

The datasets generated during and/or analyzed during the current study are available from the corresponding author on reasonable request.
