# Supplementary material for: Impact of Resolution of Hyponatremia on Neurocognitive and Motor Performance in Geriatric Patients
Source: Sci Rep. 2019 Aug 29;9:12526. doi: 10.1038/s41598-019-49054-8 (PMC6715723; doi:10.1038/s41598-019-49054-8)
Supplement: Supplementary file 1 — Supplementary Material [file 41598_2019_49054_MOESM1_ESM.docx]

**Supplemental Material**

**Impact of Resolution of Hyponatremia on**
**Neurocognitive and Motor Performance in Geriatric Patients**

Paul Thomas Brinkkoetter MD^a,¶^, Franziska Grundmann MD^a,¶^, Panteha Jazayeri Ghassabeh^a,b^, Ingrid Becker Msc^c^, Marc Johnsen MD^b^, Victor Suaréz MD^a^, Ralf-Joachim Schulz MD^b^, Thomas Streichert MD^d^ and Volker Burst MD^a^

^a^ *Department II of Internal Medicine and Center for Molecular Medicine Cologne, University of Cologne, Faculty of Medicine and University Hospital Cologne, Cologne, Germany*

*^b^ Department of Geriatric Medicine, St. Marien-Hospital, Cologne, Germany*.

^c^ *Institute of Medical Statistics and Computational Biology, University of Cologne, Cologne, Germany*

*^d^ Institute for Clinical Chemistry, University Hospital of Cologne, Cologne, Germany*

*^¶^ equal contribution*

**Item S1**

**Description of Tests included in the CGA**

*Activities of Daily Living (ADL)*

The patients’ overall functional status and the capability to perform activities of daily living (Barthel index of ADL) were assessed as described by Mahoney and Barthel. Data were provided by experienced nurses and trained technicians who had observed the patient for a maximum of 24 h. The score ranges from 0 (entirely dependent on assistance in ADL such as walking, climbing stairs, bathing, eating, and dressing) to 100 (independent) points.^27^

*Mobility and Risk of Falls -*

*Tinetti’s Performance-Oriented Mobility Assessment (TPOMA)*

TPOMA was used to assess disabilities in gait stability and balance. Patients with a TPOMA score between 20 and 24 of a maximum of 28 are at moderate risk, individuals scoring ≤19 are at high risk of falling.^28^

*Mobility and Risk of Falls - Timed Up & Go test (TuGT)*

TuGT was applied to determine impairment of mobility and gait speed. In TuGT, the time needed to raise from an armchair, walk 3 m, turn around, return to the chair and sit down is captured. A TuGT result of ≥20 s is a simple indicator of an impaired functional mobility in the elderly.^29^

*Cognition-Mini-Mental State Examination (MMSE)*

The MMSE is an 11-question comprising tool that assesses various cognitive qualities including orientation, registration, attention, calculation, recall, and language. It is a frequently utilized screening instrument for cognitive impairment with a score of 30 indicating a normal functional status and a score ≤28 indicating cognitive impairment.^30^

*Handgrip strength (HS)*

The measurement of handgrip strength assesses the maximum hand pressure of the dominant hand (or in the case of hemiplegia of the unaffected hand) and gives indications of the need for support in the ADL. Participants were asked to stand up and hold the dynamometer in the dominant hand with the arm parallel to the body. Two trials followed and the best score was used for analysis. Handgrip strength was expressed in kg.^31^

*Geriatric Depression Scale (GDS),*

The GDS (short version) was applied to screen for depressive disorders. It contains 15 items, more than 5 positive answers are indicative for depression.^32^

*Cognition - Clock-drawing Test (CDT)*

The CDT was used to assess practical cognitive skills and captures cognitive domains incompletely covered by the MMSE, in particular executive functions and visuospatial skills. A CDT score >3 is rated abnormal.^33^

*Esslinger transfer scale (ETS)*

The ETS was first described by Runge et al. in 1995. It assesses the extent of the required assistance for a safe transfer out of an armchair into a wheelchair and the degree of independence for changing positions in a bed. Stage H0 indicates complete independence, H1 indicates the need for assistance by a lay person, H2 assistance by trained lay person, H3 assistance by a health care professional, and H4 assistance of more than one health care professional. For numerical analysis the stages H0 to H4 were transformed into the numbers 0 to 4.^34^

**Item S2**

**Drugs regarded to be likely to induce or to contribute to hyponatremia in geriatric patients**

| **Category** | **Class or Generic** |
| --- | --- |
| **Anticancer** | IV cyclophosphamide |
|  | Vincristine |
| **Antidepressant** | Tricyclics |
|  | SSRI |
|  | Venlafaxine |
| **Antidiuretic** | Desmopressin |
|  | Vasopressin |
| **Antiepileptic** | Carbamazepine |
|  | Lamotrigine |
|  | Oxcarbazepine |
|  | Sodium valproate |
| **Opiate** | Fentanyl |
|  | Hydromorphone |
|  | Morphine sulfate |
|  | Oxycodone |
| **Diuretic** | Thiazides |

Abbreviations: SSRI, selective serotonin reuptake inhibitor.

**Item S3**

**Estimation of the reference range of sodium in the elderly**

Single sodium values from 355,329 patients ≥50 years stored in the database of the Institute for Clinical Chemistry of the University Hospital Cologne between 2000 and 2017 were extracted and constitute the basis for this analysis.

**a)** Estimated reference limits for plasma sodium calculated for the age groups 50 to 59 years, 60 to 69 years, 70 to 79 years, 80 to 89 years, and 90 to 99 years. Upper and lower reference limits given in the table as mEq/L. Total sample size was 355,329, the smallest strata (i.e., men between 90 and 99 years) consisted of 12,899 sodium values.

**b)** The distribution of the density function of plasma sodium in a mixed patient population can be interpreted as the superposition of the distributions of diseased and non-diseased subjects. Reference limits were estimated by separating the “central” part of the distribution, which represents the healthy population. As an example, the density function of all patients (men and women) in the age group 90 to 99 years is shown.

**
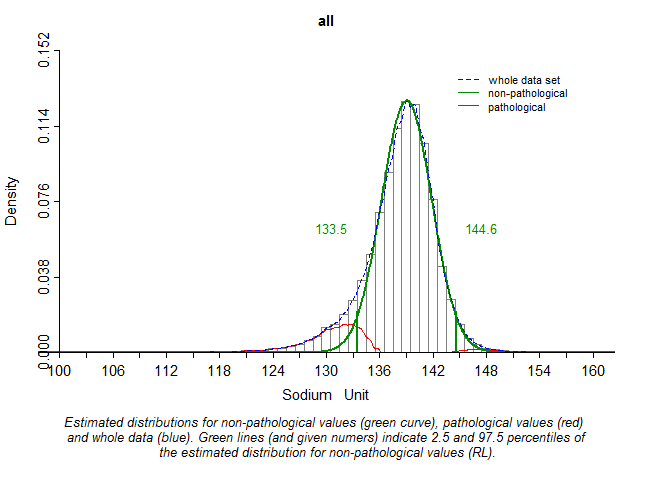
**

**Supplemental Tables**

**Supplemental Table 1:** Individual test results of CGA on admission of the primary analysis group (n=150) as well as the euvolemic, hypovolemic, and hypervolemic subgroups vs. their respective age- and gender-matched control groups, median (IQR) and mean±standard deviation

|  | **Hyponatremia** | **Control group** | **n** | **p** | |  |
| --- | --- | --- | --- | --- | --- | --- |
| **All patients** | | | | | |  |
| ADL | 50 (35-65)  49.67±22.52 | 70 (55-80)  67.87±15.88 | 150 | <0.001 | |  |
| TPOMA | 13 (8-19)  13.41±6.44 | 16 (13-22)  16.39±6.44 | 111 | 0.002 | |  |
| TuGT | 22 (17-34.25)  28.68±21.50 | 20 (15-26)  22.34±12.46 | 56 | 0.042 | |  |
| HS | 12 (1.25-18)  11.24±8.58 | 18 (12-22)  17.89±7.89 | 132 | <0.001 | |  |
| MMSE | 25 (19-27)  22.84±5.49 | 26 (23-28)  24.85±4.16 | 131 | 0.002 | |  |
| GDS | 4 (2-7)  5.16±3.55 | 3 (2-6)  4.22±3.47 | 106 | 0.065 | |  |
| CDT | 4 (3-6)  4.17±1.51 | 3 (2-4)  3.27±1.71 | 142 | <0.001 | |  |
| ETS | 1 (0-2)  1.28±1.20 | 0 (0-1)  0.58±0.72 | 141 | <0.001 | |  |
| **Euvolemic subgroup** | | | | | | |
| ADL | 45 (30-65)  46.33±23.57 | 70 (50-80)  66.60±16.42 | 75 | | <0.001 | |
| TPOMA | 12.50 (5.50-19.75)  12.73±8.00 | 16 (12.25-22)  16.38±6.92 | 56 | | 0.017 | |
| TuGT | 21.50 (18-25.75)  24.42±10.98 | 20 (13.50-26)  21.33±10.81 | 24 | | 0.495 | |
| HS | 11 (0-17)  10.97±8.34 | 18 (13-22)  17.54±8.03 | 63 | | <0.001 | |
| MMSE | 23 (19-26.25)  22.38±4.89 | 25 (23-27.25)  24.64±3.93 | 66 | | 0.010 | |
| GDS | 4 (2-7)  5.17±3.65 | 3 (2-5)  4.28±3.66 | 54 | | 0.186 | |
| CDT | 4 (3-6)  4.25±1.44 | 3 (2-4)  3.11±1.28 | 73 | | <0.001 | |
| ETS | 1 (0-2)  1.28±1.17 | 0 (0-1)  0.54±0.74 | 69 | | <0.001 | |
| **Hypovolemic subgroup** | | | | | | |
| ADL | 55 (35-70)  54.36±22.16 | 75 (65-85)  71.49±14.25 | 47 | | <0.001 | |
| TPOMA | 15 (11-19)  14.14±7.24 | 17 (13-21.75)  16.67±5.65 | 36 | | 0.039 | |
| TuGT | 31.50 (16.75-40)  35.23±30.86 | 20.50 (15-30.25)  24.45±15.70 | 22 | | 0.079 | |
| HS | 11 (2-18)  11.38±9.10 | 17 (11.75-21)  16.36±5.86 | 42 | | 0.012 | |
| MMSE | 27 (20.50-28)  23.70±6.60 | 26 (24.25-28.75)  25.50±4.12 | 40 | | 0.192 | |
| GDS | 4 (2-6)  3.88±3.10 | 3 (2-5.25)  3.76±3.02 | 34 | | 0.993 | |
| CDT | 4 (3-6)  4.02±1.61 | 3 (2-4)  3.53±2.45 | 43 | | 0.049 | |
| ETS | 1 (0-2)  1.24±1.13 | 0 (0-1)  0.56±0.62 | 45 | | <0.001 | |
| **Hypervolemic subgroup** | | | | | | |
| ADL | 50 (31.25-67.50)  50.71±19.18 | 62.50 (55-80)  65.18±16.47 | 28 | | 0.006 | |
| TPOMA | 14 (9-20)  14.00±7.52 | 15 (12-22)  15.89±6.71 | 19 | | 0.506 | |
| TuGT | 22 (15-30.50)  24.50±11.10 | 19.50 (14.50-25.25)  20.10±7.51 | 10 | | 0.332 | |
| HS | 14 (0-19)  11.67±8.66 | 20 (12-26)  21.11±9.53 | 27 | | 0.002 | |
| MMSE | 24 (18.50-27)  22.68±5.11 | 26 (22-28)  24.40±4.83 | 25 | | 0.310 | |
| GDS | 7.50 (5-10.25)  7.56±2.83 | 3 (2-7)  4.89±3.76 | 18 | | 0.033 | |
| CDT | 4 (3-6)  4.19±1.55 | 3.5 (2-4)  3.27±1.25 | 26 | | 0.028 | |
| ETS | 1 (0-2)  1.37±1.42 | 1 (0-1)  0.74±0.81 | 27 | | 0.079 | |

Numbers are dimensionless except TuGT (s) and HS (kg)

**Supplemental Table 2:** Individual test results of CGA on admission of the primary analysis group (n=150) as well as the euvolemic, hypovolemic, and hypervolemic subgroups vs. their respective matched reference groups, median (IQR) and mean±standard deviation

|  | **Hyponatremia** | **Reference group** | **n** | **p** | |  |
| --- | --- | --- | --- | --- | --- | --- |
| **All patients** | | | | | |  |
| ADL | 50 (35-65)  49.67±22.52 | 50 (35-65)  49.67±22.52 | 150 | 1.000 | |  |
| TPOMA | 13.5 (8-19)  14.55±7.65 | 14 (10-20)  14.85±8.02 | 66 | 0.795 | |  |
| TuGT | 22 (17-35.25)  27.94±14.22 | 23 (15-30)  22.83±12.73 | 66 | 0.126 | |  |
| HS | 12 (1-18)  11.14±8.71 | 11 (0-16)  10.70±9.78 | 137 | 0.417 | |  |
| MMSE | 25 (19-27)  22.94±5.21 | 24 (20-27)  23.76±4.27 | 117 | 0.201 | |  |
| GDS | 4 (2-7)  5.18±3.47 | 4 (2-6.5)  4.59±3.38 | 87 | 0.259 | |  |
| CDT | 4 (3-6)  4.14±1.52 | 4 (3-6)  4.08±1.48 | 147 | 0.684 | |  |
| ETS | 1 (0-2)  1.28±1.20 | 1 (0-2)  1.43±1.21 | 141 | 0.248 | |  |
| **Euvolemic subgroup** | | | | | | |
| ADL | 45 (30-65)  46.33±23.57 | 45 (30-65)  46.33±23.57 | 75 | | 1.000 | |
| TPOMA | 13 (6-19)  14.48±8.38 | 12 (5.25-20.75)  14.06±8.57 | 31 | | 0.773 | |
| TuGT | 21.5 (17.75-26.50)  22.56±7.63 | 25 (15.75-34)  23.19±12.29 | 16 | | 0.820 | |
| HS | 11 (0-16.50)  10.55±8.36 | 11 (0-17)  11.62±10.54 | 69 | | 0.662 | |
| MMSE | 23 (19-26.25)  22.52±4.85 | 24 (20-27)  23.74±4.41 | 61 | | 0.105 | |
| GDS | 4 (2-7)  4.86±3.73 | 4 (2-5)  4.23±3.34 | 43 | | 0.394 | |
| CDT | 4 (3-6)  4.17±1.49 | 4 (3-6)  4.08±1.45 | 75 | | 0.519 | |
| ETS | 1 (0-2)  1.28±1.17 | 1 (0.75-2.25)  1.48±1.21 | 69 | | 0.228 | |
| **Hypovolemic subgroup** | | | | | | |
| ADL | 55 (35-70)  54.36±22.16 | 55 (35-70)  54.36±22.16 | 47 | | 1.000 | |
| TPOMA | 15 (11-19)  14.67±6.58 | 16 (10-22.25)  17.24±7.41 | 21 | | 0.204 | |
| TuGT | 31 (16-40)  33.00±18.44 | 19 (12.50-25.75)  19.64±7.81 | 11 | | 0.045 | |
| HS | 12 (2-18)  11.93±9.36 | 10 (0-15)  9.24±9.06 | 42 | | 0.102 | |
| MMSE | 27 (20.50-28)  24.00±5.86 | 24 (22-27)  23.44±4.18 | 32 | | 0.369 | |
| GDS | 4 (2-6)  4.46±2.97 | 4 (1.50-6)  4.88±3.86 | 26 | | 0.547 | |
| CDT | 4 (3-6)  4.05±1.63 | 4 (3-6)  4.20±1.49 | 44 | | 0.568 | |
| ETS | 1 (0-2)  1.24±1.13 | 1 (0-2)  1.38±1.23 | 45 | | 0.544 | |
| **Hypervolemic subgroup** | | | | | | |
| ADL | 50 (31.25-67.50)  50.71±19.18 | 50 (31.25-67.50)  50.71±19.18 | 28 | | 1.000 | |
| TPOMA | 14 (9-20)  14.50±7.96 | 15 (8.75-19)  13.00±7.29 | 14 | | 0.753 | |
| TuGT | 22 (16-36.50)  31.75±15.77 | 24 (18-32)  26.50±18.56 | 8 | | 0.398 | |
| HS | 13.50 (0.50-18.50)  11.42±8.77 | 11.50 (0-20)  10.62±8.76 | 26 | | 0.639 | |
| MMSE | 24 (18.50-27)  22.58±5.20 | 25 (19-27)  24.25±4.16 | 24 | | 0.148 | |
| GDS | 7 (5-10)  7.00±3.01 | 5 (3-7.50)  5.00±2.79 | 18 | | 0.059 | |
| CDT | 4 (3.25-6)  4.18±1.49 | 4 (3-5.75)  3.89±1.60 | 28 | | 0.388 | |
| ETS | 1 (0-2)  1.37±1.42 | 1 (0-2.75)  1.37±1.21 | 27 | | 0.969 | |

Numbers are dimensionless except TuGT (s) and HS (kg)

**Supplemental Table 3:** Change (Δ) in test results between admission and discharge (positive values indicate an improvement) in the primary analysis group and matched reference group, median (IQR) and mean±SD

|  | **Hyponatremia** | **n** | **p** | **Reference group** | **n** | **p** |
| --- | --- | --- | --- | --- | --- | --- |
| ΔADL | 10 (0-25)  14.31±17.12 | 150 | <0.001 | 5 (0-15)  9.84±14.67 | 150 | <0.001 |
| ΔTPOMA | 4 (1-8)  5.40±7.67 | 104 | <0.001 | 4 (0-7)  4.56±5.82 | 71 | <0.001 |
| ΔTuGT [s] | 5 (1-7)  4.27±13.37 | 86 | 0.002 | 5 (2-5)  4.59±5.22 | 78 | <0.001 |
| ΔHS [kg] | 0 (0-3)  1.41±6.45 | 127 | 0.007 | 0 (0-2)  0.72±5.15 | 131 | 0.008 |
| ΔMMSE | 0 (0-3)  1.43±3.71 | 118 | <0.001 | 0 (0-1)  0.72±2.17 | 125 | <0.001 |
| ΔGDS | 0 (0-1)  0.09±2.11 | 96 | 0.364 | 0 (0-0.25)  0.03±1.79 | 106 | 0.204 |
| ΔCDT | 0 (0-0)  -0.22±1.20 | 135 | 0.032 | 0 (0-0)  -0.21±0.88 | 140 | 0.009 |
| ΔETS | 0 (0-1)  0.42±0.95 | 126 | 0.248 | 0 (0-1)  0.59±1.00 | 139 | <0.001 |

Numbers are dimensionless except ΔTuGT (s) and ΔHS (kg)

**Supplemental Table 4a:** Change (Δ) in test results between admission and discharge (positive values indicate an improvement) in effectively treated (i.e., Δ[Na^+^] >5mEq/L, n=108) and ineffectively treated (i.e., Δ[Na^+^] ≤5mEq/L, n=42) hyponatremic patients of the primary analysis group vs. matched reference group, median (IQR) and mean±SD

|  | **Δ[Na^+^] >5mEq/L** | **Reference group** | **n** | **p** |
| --- | --- | --- | --- | --- |
| ΔADL | 10 (0-23.75)  14.51±18.08 | 5 (0-10)  9.73±15.19 | 108 | 0.014 |
| ΔTPOMA | 4 (2-8)  7.12±10.93 | 5 (0-7)  3.94±6.15 | 33 | 0.091 |
| ΔTuGT [s] | 5 (1.75-6.25)  1.95±13.73 | 5 (2-5)  5.11±6.88 | 38 | 0.389 |
| ΔHS [kg] | 0 (0-3)  1.81±6.94 | 0 (0-1.50)  1.47±5.17 | 77 | 0.975 |
| ΔMMSE | 0 (0-3)  1.67±4.31 | 0 (0-1)  0.81±2.17 | 75 | 0.007 |
| ΔGDS | 0 (0-1)  0.57±2.13 | 0 (0-0)  -0.17±2.33 | 53 | 0.712 |
| ΔCDT | 0 (0-0)  -0.12±1.17 | 0 (0-0)  -0.20±0.76 | 91 | 0.757 |
| ΔETS | 0 (0-1)  0.46±0.97 | 0 (0-1)  0.56±0.90 | 84 | 0.449 |
|  |  |  |  |  |
|  | **Δ[Na^+^] ≤5mEq/L** | **Reference group** | **n** | **p** |
| ΔADL | 10 (0-26.25)  13.81±14.52 | 7.5 (0-15)  10.12±13.41 | 42 | 0.063 |
| ΔTPOMA | 3 (0-8)  4.59±5.95 | 2 (0-9.25)  2.65±5.20 | 17 | 0.345 |
| ΔTuGT [s] | 5 (0-7)  9.21±16.34 | 4 (2-5)  3.14±2.21 | 14 | 0.234 |
| ΔHS [kg] | 0 (-1-2)  0.5±5.50 | 0 (0-2)  -0.44±5.60 | 32 | 0.705 |
| ΔMMSE | 0 (0-0.25)  1.16±2.58 | 0 (0-1)  0.44±1.36 | 25 | 0.168 |
| ΔGDS | 0 (0-1)  0.20±1.15 | 0 (0-1)  0.53±0.83 | 15 | 0.343 |
| ΔCDT | 0 (0-0)  -0.44±1.24 | 0 (-1-0)  -0.24±1.13 | 34 | 0.480 |
| ΔETS | 0 (0-1)  0.27±0.94 | 0 (0-1)  0.48±1.09 | 33 | 0.435 |

Numbers are dimensionless except ΔTuGT (s) and ΔHS (kg)

**Supplemental Table 4b:** Change (Δ) in test results between admission and discharge (positive values indicate an improvement) in effectively (i.e., Δ[Na^+^] >5mEq/L, n=55) and ineffectively (i.e., Δ[Na^+^] ≤5mEq/L, n=20) treated hyponatremic patients of the euvolemic subgroup vs. matched reference group, median (IQR) and mean±SD

|  | **Δ[Na^+^] >5mEq/L** | **Reference group** | **n** | **p** |
| --- | --- | --- | --- | --- |
| ΔADL | 10 (0-25)  15.55±19.38 | 5 (0-20)  11.09±17.37 | 55 | 0.166 |
| ΔTPOMA | 4 (2-8)  6.29±4.08 | 5 (1.50-7.25)  3.36±6.79 | 14 | 0.148 |
| ΔTuGT [s] | 5 (0-5)  3.06±10.73 | 5 (2-7)  5.81±8.91 | 16 | 0.330 |
| ΔHS [kg] | 1 (0-3.50)  3.31±6.89 | 0 (0-2)  2.05±6.58 | 39 | 0.293 |
| ΔMMSE | 1 (0-3.5)  1.53±5.22 | 0 (0-1)  0.83±2.52 | 36 | 0.008 |
| ΔGDS | 0 (0-0)  -0.48±2.23 | 0 (0-0)  -0.26±2.86 | 23 | 0.504 |
| ΔCDT | 0 (0-0)  0.16±1.19 | 0 (0-0)  -0.21±0.71 | 43 | 0.156 |
| ΔETS | 0 (0-1)  0.24±1.01 | 1 (0-1)  0.76±0.89 | 37 | 0.015 |
|  |  |  |  |  |
|  | **Δ[Na^+^] ≤5mEq/L** | **Reference group** | **n** | **p** |
| ΔADL | 12.50 (0-30)  15.50±16.34 | 5 (0-18.75)  11.75±15.50 | 20 | 0.284 |
| ΔTPOMA | 2.50 (0-8.75)  5.00±7.62 | 3 (0-10)  3.22±6.40 | 9 | 0.726 |
| ΔTuGT [s] | 4.50 (-1.50-6)  0.00±4.90 | 4.50 (1-5.75)  3.50±2.95 | 6 | 0.104 |
| ΔHS [kg] | 0 (-2.25-2.50)  -0.06±4.61 | 0 (0-1.25)  -0.31±3.96 | 16 | 0.887 |
| ΔMMSE | 0.50 (0-1.25)  1.64±3.30 | 0 (0-1)  1.21±1.25 | 14 | 0.058 |
| ΔGDS | 0 (0-1.50)  0.38±1.51 | 0 (0-0.75)  0.63±0.92 | 8 | 0.713 |
| ΔCDT | 0 (-1-0)  -0.67±1.24 | 0 (0-0)  0.00±1.28 | 18 | 0.144 |
| ΔETS | 0 (0-1)  0.47±0.62 | 0 (0-1)  0.47±0.87 | 17 | 0.951 |

Numbers are dimensionless except ΔTuGT (s) and ΔHS (kg)

**Supplemental Table 4c:** Change (Δ) in ADL and MMSE results between admission and discharge (positive values indicate an improvement) in effectively (i.e., Δ[Na^+^] >5mEq/L) treated hyponatremic patients of the euvolemic subgroup after removal of cases that were treated by withdrawal of both thiazide diuretics or other HN-inducing drugs (1) as well as withdrawal on thiazides (2) or other HN-inducing drugs (3) vs. matched reference group, median (IQR) and mean ± SD

|  | **Δ[Na^+^] >5mEq/L** | **Reference group** | **n** | **p** |
| --- | --- | --- | --- | --- |
| **ΔADL** |  |  |  |  |
| 1 | 10 (0-25)  15.73 ± 20.90 | 5 (0-25)  13.41 ± 18.99 | 41 | 0.584 |
| 2 | 10 (0-17.5)  12.50 ± 17.26 | 5 (0-11.25)  9.40 ± 17.15 | 42 | 0.274 |
| 3 | 5 (0-22.5)  11.25 ± 18.59 | 5 (0-17.5)  11.96 ± 19.74 | 28 | 0.987 |
|  |  |  |  |  |
| **ΔMMSE** |  |  |  |  |
| 1 | 1 (0-3.75)  2.14 ± 2.97 | 0 (0-1)  0.77 ± 2.94 | 24 | 0.015 |
| 2 | 5 (0-3)  0.85 ± 4.97 | 0 (0-1)  0.58 ± 2.62 | 30 | 0.017 |
| 3 | 1 (0-3)  1.52 ± 2.40 | 0 (0-1)  0.56 ± 2.83 | 18 | 0.041 |
|  |  |  |  |  |

Numbers are dimensionless.

**Supplemental Table 4d:** Change (Δ) in test results between admission and discharge (positive values indicate an improvement) in effectively (i.e., Δ[Na^+^] >5mEq/L, n=33) and ineffectively (i.e., Δ[Na^+^] ≤5mEq/L, n=14) treated hyponatremic patients of the hypovolemic subgroup vs. matched reference group, median (IQR) and mean ± SD

|  | **Δ[Na^+^] >5mEq/L** | **Reference group** | **n** | **p** |
| --- | --- | --- | --- | --- |
| ΔADL | 10 (0-20)  12.33 ± 17.15 | 5 (0-10)  8.36 ± 12.56 | 33 | 0.159 |
| ΔTPOMA | 5 (3-8)  4.81 ± 3.19 | 4 (0-9.25)  3.09 ± 3.62 | 11 | 0.240 |
| ΔTuGT [s] | 5 (5-10)  5.83 ± 3.35 | 5 (1.5-5)  3.92 ± 2.02 | 12 | 0.183 |
| ΔHS [kg] | 0 (-0.50-2.25)  0. ± 4.53 | 0 (0-0)  0.87 ± 3.18 | 24 | 0.569 |
| ΔMMSE | 0 (0-1.75)  1.76 ± 3.70 | 0 (0-2.25)  0.72 ± 2.07 | 25 | 0.476 |
| ΔGDS | 0 (0-1)  0.15 ± 2.00 | 0 (0-1)  -0.25 ± 2.27 | 20 | 0.527 |
| ΔCDT | 0 (0-0)  -0.52 ± 1.21 | 0 (0-0)  -0.17 ± 0.97 | 29 | 0.235 |
| ΔETS | 0 (1-1)  0.67 ± 0.92 | 0 (0-1)  0.33 ± 0.99 | 30 | 0.247 |
|  |  |  |  |  |
|  | **Δ[Na^+^] ≤5mEq/L** | **Reference group** | **n** | **p** |
| ΔADL | 10 (0-25)  12.50 ± 12.82 | 10 (0-15)  9.26 ± 11.41 | 14 | 0.173 |
| ΔTPOMA | 7 (2.50-8.50)  6.60 ± 2.07 | 2 (0-5)  2.80 ± 4.66 | 5 | 0.223 |
| ΔTuGT [s] | 6 (2.50-22)  19.50 ± 20.90 | 3 (2.50-5)  3.17 ± 1.83 | 6 | 0.058 |
| ΔHS [kg] | 0 (-1-6)  1.36 ± 7.72 | 0 (-0.25-1.25)  -1.64 ± 6.50 | 11 | 0.386 |
| ΔMMSE | 1.50 (0-3.75)  1.50 ± 1.29 | 1 (0-4)  1.50 ± 2.38 | 4 | 1.000 |
| ΔGDS | 0 (-1.75-1)  0.00 ± 1.00 | 0 (0-1)  1.00 ± 1.00 | 3 | 0.276 |
| ΔCDT | 0 (-1.50-0)  -0.33 ± 1.66 | 0 (-2-0)  -0.78 ± 1.09 | 9 | 0.414 |
| ΔETS | 0 (0-1)  0.00 ± 1.58 | 0 (0-2)  0.44 ± 1.74 | 9 | 0.458 |

Numbers are dimensionless except ΔTuGT (s) and ΔHS (kg)

**Supplemental Table 4e:** Change (Δ) in test results between admission and discharge (positive values indicate an improvement) in effectively (i.e., Δ[Na^+^] >5mEq/L, n=20) and ineffectively (i.e., Δ[Na^+^] ≤5mEq/L, n=8) treated hyponatremic patients of the hypervolemic subgroup vs. matched reference group, median (IQR) and mean ± SD

|  | **Δ[Na^+^] >5mEq/L** | **Reference group** | **n** | **p** |
| --- | --- | --- | --- | --- |
| ΔADL | 10 (1.25-20)  15.25 ± 16.42 | 2.5 (0-10)  8.25 ± 12.90 | 20 | 0.066 |
| ΔTPOMA | 3 (0.50-5.50)  11.75 ± 21.59 | 4 (2-7)  6.13 v 7.85 | 8 | 0.865 |
| ΔTuGT [s] | 5 (-6-5.5)  -4.50 ± 22.38 | 5 (0-5)  5.40 ± 7.40 | 10 | 0.263 |
| ΔHS [kg] | 0 (-1.75-0)  0.21 ± 9.63 | 0 (0-1.25)  0.86 ± 3.18 | 14 | 0.423 |
| ΔMMSE | 0.50 (0-4)  1.86 ± 2.63 | 0 (0-2.25)  0.93 ± 3.18 | 14 | 0.190 |
| ΔGDS | 0 (0-2)  1.10 ± 1.85 | 0 (0-0)  0.20 ± 0.42 | 10 | 0.131 |
| ΔCDT | 0 (0-0)  -0.16 ± 0.90 | 0 (0-0)  -0.21 ± 0.54 | 19 | 0.730 |
| ΔETS | 0 (0-1.25)  0.59 ± 0.94 | 1 (0-1)  0.53 ± 0.62 | 17 | 0.794 |
|  |  |  |  |  |
|  | **Δ[Na^+^] ≤5mEq/L** | **Reference group** | **n** | **p** |
| ΔADL | 7.5 (0-27.50)  11.88 ± 13.61 | 5 (0-18.75)  7.50 ± 11.95 | 8 | 0.336 |
| ΔTPOMA | 0 (-1-2)  0.00 ± 0.00 | 1 (0-2)  0.67 ± 1.15 | 3 | 0.317 |
| ΔTuGT [s] | 5 (-10-10.50)  6.00 ± 1.41 | 2 (0.50-4.25)  2.00 ± 0.00 | 2 | 0.180 |
| ΔHS [kg] | 0 (0-1)  0.40 ± 1.52 | 1 (-2.75-8.50)  1.80 ± 8.26 | 5 | 0.715 |
| ΔMMSE | 0 (0-0)  0.00 ± 0.00 | 0 (0-0.75)  0.29 ± 0.49 | 7 | 0.157 |
| ΔGDS | 0 (0-0)  0.00 ± 0.00 | 0 (0-1)  0.00 ± 0.00 | 4 | 1.000 |
| ΔCDT | 0 0-0)  0.00 ± 0.00 | 0 (0-0)  -0.14 ± 0.38 | 7 | 0.317 |
| ΔETS | 0 (0-0)  0.14 ± 0.38 | 0.5 (0-1)  0.57 ± 0.53 | 7 | 0.083 |

Numbers are dimensionless except ΔTuGT (s) and ΔHS (kg)

**Supplemental Table 5:** Change (Δ) in test results between admission and discharge (positive values indicate an improvement) in patients of the primary analysis group with 125 ≤[Na^+^] <130 mEq/L (n=103) and with [Na^+^] <125 mEq/L (n=47) at baseline (i.e., on admission) vs. matched reference group, median (IQR) and mean±SD.

|  | **[Na^+^] <130 mEq/L** | **Reference group** | **n** | **p** |
| --- | --- | --- | --- | --- |
| ΔADL | 10 (0-25) | 5 (0-15) | 103 | 0.011 |
|  | 14.42±16.20 | 9.85±14.18 |  |  |
| ΔTPOMA | 4 (1- 8) | 2(0-7) | 36 | 0.071 |
|  | 5.41±8.42 | 4.24±6.66 |  |  |
| ΔTuGT [s] | 5 (2-7.25) | 5 (5-5) | 33 | 0.465 |
|  | 6.11±12.51 | 4.31±4.67 |  |  |
| ΔHS [kg] | 0 (0-2.25) | 0 (0-2) | 71 | 0.650 |
|  | 1.36±5.87 | 0.45±4.74 |  |  |
| ΔMMSE | 0 (0-3) | 0 (0-1) | 64 | <0.001 |
|  | 1.81±3.09 | 0.33±1.93 |  |  |
| ΔGDS | 0 (0-1) | 0 (0-0.50) | 41 | 0.818 |
|  | 0.16±2.52 | -0.06±0.93 |  |  |
| ΔCDT | 0 (0-0) | 0 (0-0) | 87 | 1.000 |
|  | - 0.23±1.26 | -0.24±0.93 |  |  |
| ΔETS | 0 (0-1) | 0 (0-1) | 79 | 0.145 |
|  | 0.28±0.95 | 0.58±1.05 |  |  |
|  |  |  |  |  |
|  | **[Na^+^] <125 mEq/L** | **Reference group** | **n** | **p** |
| ΔADL | 10 (0-15) | 5 (0-10) | 47 | 0.077 |
|  | 14.09±19.16 | 9.81±15.85 |  |  |
| ΔTPOMA | 4 (2- 8.50) | 5(2-8.25) | 14 | 0.575 |
|  | 5.38±5.40 | 5.16±4.00 |  |  |
| ΔTuGT [s] | 5 (-5.25-5.75) | 5 (2-5) | 19 | 0.255 |
|  | -0.50±14.57 | 5.03±6.06 |  |  |
| ΔHS [kg] | 0 (0-3.50) | 0 (0-1) | 38 | 0.910 |
|  | 1.59±7.61 | 0.45±4.74 |  |  |
| ΔMMSE | 0 (0-2) | 0 (0-1) | 36 | 0.890 |
|  | 0.67±4.68 | 1.25±5.89 |  |  |
| ΔGDS | 0 (0-0) | 0 (0-0.50) | 27 | 0.722 |
|  | -0.03±1.10 | 0.19±2.00 |  |  |
| ΔCDT | 0 (0-0) | 0 (0-0) | 38 | 0.672 |
|  | - 0.21±1.08 | -0.14±0.89 |  |  |
| ΔETS | 1 (0-1) | 0.50 (0-1) | 38 | 0.814 |
|  | 0.71±0.90 | 0.61±0.89 |  |  |

Numbers are dimensionless except ΔTuGT (s) and ΔHS (kg).
